# Feasibility of perioperative remote monitoring of patient‐generated health data in complex surgical oncology

**DOI:** 10.1002/jso.27106

**Published:** 2022-09-28

**Authors:** Laleh G. Melstrom, Xiaoke Zhou, Andreas Kaiser, Kevin Chan, Clayton Lau, Mustafa Raoof, Susanne G. Warner, Ali Zhumkhawala, Bertram Yuh, Gagandeep Singh, Yuman Fong, Virginia Sun

**Affiliations:** ^1^ Department of Surgery City of Hope Division of Surgical Oncology Duarte California USA; ^2^ Department of Population Sciences City of Hope Duarte California USA; ^3^ Department of Surgery City of Hope Division of Colorectal Surgery Duarte California USA; ^4^ Department of Surgery City of Hope Division of Urologic Oncology Duarte California USA; ^5^ Department of Surgery Mayo Clinic Rochester Minnesota USA

**Keywords:** patient‐generated health data, patient‐reported outcomes, telemonitoring

## Abstract

**Background:**

The feasibility of remote perioperative telemonitoring of patient‐generated physiologic health data and patient‐reported outcomes in a high risk complex general and urologic oncology surgery population is evaluated.

**Methods:**

Complex general surgical/urologic oncology patients wore a pedometer, completed ePROs (electronic patient‐reported outcome surveys) and record their vitals (weight, pulse, pulse oximetry, blood pressure, and temperature) via a telehealth app platform. Feasibility (% adherence) was assessed as the primary outcome measure.

**Results:**

Twenty‐one patients with a median age 58 (32−82) years were included. The readmission rate was 33% and the incidence of ≥Grade 3a morbidity was 24%. Adherence to vital sign and ePRO measurements was 95% before surgery, 91% at discharge, and 82%, 68%, and 64% at postdischarge d2, 7, 14, and 30, respectively. There was significant worsening of mobility, self‐care and usual daily activity at postdischarge d2 compared to preoperative baseline (*p* < 0.05). Median daily preoperative steps taken by patients with <Grade 3a versus ≥Grade 3a postoperative morbidity was 6062 versus 4166 (*p* < 0.05). Of those interviewed, 87% (13/15) viewed vital sign devices as helpful in recovery.

**Conclusions:**

Telemonitoring is feasible in a general surgical and urologic oncology setting. Future studies will ascertain optimal patient selection, duration, and extent of perioperative monitoring.

## 1 INTRODUCTION

Surgeons and cancer centers are increasingly asked to provide evidence of the quality and value of their care. This paradigm shift is occurring at a time where tremendous advances in surgical techniques are taking place. The advent of minimally invasive surgical techniques has resulted in a shift in the way surgical teams care for patients postoperatively. Patients are now discharged earlier and earlier after surgery, with postoperative recovery primarily taking place at home. Postoperative complications that traditionally arise in the hospital are now developing, potentially unnoticed, at home and in the outpatient setting.

Outcomes that are used to measure quality surgical oncology care are also evolving, with efforts to focus on more patient‐centered variables. Historically, medical and surgical outcomes are measured by disease‐ and systems‐related parameters like length of hospital stay, morbidity, readmission rates, and mortality.^1^, ^2^ While important, these measures may not accurately reflect the surgical care experience from a patient's perspective.

A promising approach to modernize perioperative care and improve patient‐centeredness is through remote monitoring of patient‐generated health data (PGHD). In the United States, the Office of the National Coordinator for Health Information Technology defines PGHDs as “health‐related data created, recorded, or gathered by or from patients (or family members or other caregivers) to help address a health concern.”^3^ PGHDs may include health history, treatment history, biometric data, symptoms, lifestyle choices. Importantly, patients are responsible for capturing these data, which is distinct from traditional data generated in clinical settings.^3^ PGHDs are increasingly being used in routine cancer care as quality and value indicators,^2^, ^4^, ^5^, ^6^ with robust evidence on its impact on clinical outcomes in advanced cancer populations.^7^ Research on PGHDs and remote monitoring in surgical oncology is somewhat nascent, with the majority of the current evidence focused on electronic monitoring of symptoms.^8^ Knowledge gaps remain on PGHD's utility and impact on surgical outcomes/care decision‐making, particularly for data captured remotely through wearables and other devices.

Our research team had previously conducted a proof‐of‐concept study to assess the feasibility and acceptability of electronic symptom and functional status monitoring in major abdominal cancer surgery.^9^ We found that remote monitoring was feasible and acceptable, and exploratory analysis suggest that the number of daily steps may be associated with postoperative complications. The original proof‐of‐concept study above did not include remote capture and monitoring of patient‐generated physiologic/biometrics data such as vital signs. Therefore, in the present study we conducted a second proof‐of‐concept feasibility trial of remote perioperative telemonitoring that combines objectively measured physiologic data (vital signs and daily steps) and electronic patient‐reported outcomes (ePROs) in a complex surgical oncology and urologic oncology setting.

## 2 METHODS

This was a proof‐of‐concept trial that aimed to assess (1) the feasibility of remote monitoring of combined objective/subjective PGHDs/ePROs in surgical/urologic oncology, and (2) to explore the trajectory of the PGHDs overtime, from before surgery to after surgery.

Patients eligible for participation in the study were scheduled to undergo a curative resection for urologic (kidney and bladder) or GI cancers (gastric, colorectal, and peritoneal surface malignancy, liver and pancreas), were >18 years old and English speaking. Between August and December 2020, eligible patients who met the study inclusion criteria were identified and recruited from the surgical and urologic oncology ambulatory clinics of a National Cancer Institute‐designated comprehensive cancer center. The Institutional Review Board (IRB19040) approved study procedures and all participating patients provided written informed consent before enrollment. The study was registered at clinicaltrials.gov (NCT04501913) and was Health Insurance Portability and Accountability Act (HIPAA) compliant.

### 2.1 Remote monitoring and outcomes thresholds design

Following informed consent, patients were provided with the following: (1) Federal and Drug Administration (FDA)‐cleared blue‐tooth enabled devices from the company mTelehealth (thermometer, digital weight scale, sphygmometer, pulse oximeter) for the capture of vital signs; (2) a commercially available wristband pedometer (Vivofit 4; Garmin Ltd) for tracking functional recovery (daily steps); and (3) a study tablet with HIPAA compliant Aetonix A Touch Away™ mobile application platform. The authors have no financial relationships with the third‐party vendors and their products used in the study. The FDA‐cleared devices for vital signs were paired with the study tablet, and assessments of ePROs were also captured through the tablet. Trained research staff assisted patients with setup and provided instructions on device/engagement platform use.

Thresholds for all PGHDs were predetermined to guide actions based on the data that patients provided. Vital sign thresholds included: (1) weight increase/decrease of 2 kg from discharge; (2) temperature >38°C with heart rate >110 or systolic blood pressure <90 or >180 or a temperature of >38.3°C independent of other vital signs; (3) oxygen saturation <90%; 4) heart rate >110 beats per min (>120 if last heart rate at discharge was >100); (5) systolic blood pressure less than 90 (<85 if last systolic blood pressure at discharge was <100) or >180. Functional status threshold was based on our first proof‐of‐concept trial and set at daily steps count of <1500. For the ePROs (symptoms/Quality of life [QOL]), thresholds were set at one or more symptoms/QOL items with a moderate to severe intensity score (4 or higher).

### 2.2 Outcome measures

Feasibility was assessed by (1) overall accrual, and attrition rates and (2) patient's ability to use the remote perioperative monitoring equipment.

The study included three brief measures to assess ePROs. Symptom severity and symptom interference with activities were assessed using the MD Anderson Symptom Inventory (MDASI), a validated measure of 13 common cancer‐related symptoms as rated on a 10‐point scale.^10^ The EuroQol 5‐dimensional descriptive system (EQ‐5D‐5L) was used to assess quality of life and general health status.^11^ This validated instrument functions to evaluate the following 5 QOL variables: mobility, self‐care, usual activities, pain or discomfort, and anxiety or depression. Overall health state using a visual analog scale with end points labeled. “best to worst imaginable” health state (range, 0−100) was used as a final overall metric. The EQ‐5D‐5L instrument has been widely employed in quality‐adjusted survival analyses and clinical trials.^11^, ^12^, ^13^ Finally, the Patient‐Reported Outcomes Measurement Information System (PROMIS) General Physical and Mental Health‐Short From was used to assess physical and mental health status.^14^ The four items are scored on a 5‐point Likert scale: 1 (poor); 2 (fair); 3 (good); 4 (very good); and 5 (excellent).

Relevant surgical and other clinical data were obtained via the electronic health record, including surgery date, comorbidities, primary diagnosis, procedure type, surgical technique (open, laparoscopic, or robot assisted), American Society of Anesthesiologists' (ASA) classification, length of hospital stay, and readmissions. Each patient's performance status was evaluated utilizing the Eastern Cooperative Oncology Group performance scale score ranging from 0 to 5, where 0 denotes no symptoms and 5 indicates death. Postoperative complications were calculated using the Comprehensive Complication Index (CCI) based on the Clavien−Dindo classification.^15^, ^16^

### 2.3 Study procedures

All patients were consented at least 3−7 days before surgery. This design was included to capture preop/baseline data on all outcomes, and also provided ample time for device setup and instructions. All patients completed baseline assessments of vital signs and ePROs electronically before surgery. Patients were instructed to bring their Vivofit pedometer and the study tablet to their hospital admissions. Outcomes were collected again postoperatively at hospital discharge and at Days 2, 7, 14, and 30 postdischarge. For the at hospital discharge time point, we used the last set of inpatient data recorded; this design was included so patients did not need to bring the FDA‐cleared devices to the hospital. At each of the postdischarge time points, patients received a reminder through their study tablet to provide a set of vital signs and complete ePROs. Daily steps data were continuously collected 3−7 days before surgery, during hospitalization and up to 30 days postdischarge.

When PGHDs deviated from the predetermined thresholds, an alert via the Aetonix application was automatically generated within 1 min to trained Research Nurses. The alerts prompted the Research Nurses to proactively contact patients via telephone for further assessments, triage, and surgical team notification. Standard institutional triage nursing protocols were followed. Each encounter prompted by an alert were documented as an encounter note in the electronic medical records.

At the final assessment time point (Day 30 postdischarge), patients completed a brief survey to assess acceptability and overall satisfaction with the monitoring. Patients provided feedback on the following: (1) use of devices and mobile application; (2) items in electronic surveys that were distressing or challenging to understand; (3) length of surveys and point of administration; and (4) items that were not covered but should be considered. After the study was completed patients also participated in an exit interview with questions regarding feedback on useful aspects of the telemonitoring program and devices in functional recovery and communication with their surgical team.

### 2.4 Statistical analysis

Vital signs and ePROs were wirelessly captured through the study tablet, synchronized for feedback system/alert purposes, and transferred to a study‐specific REDCap database. Vivofit daily steps data were wirelessly and automatically transferred to the same REDCap database.

Data were summarized using means, medians, standard deviations, minimum and maximum for continuous data, and proportions and percentages for categorical data. For PGHD alerts, every instance of Research Nurse initiated telephone assessments was considered a monitoring encounter, and the total number of monitoring encounters was recorded.

Associations determined in this study were exploratory. A linear regression was performed between the number of daily steps at Day 14 and postoperative complications as calculated by the CCI and a correlation coefficient was calculated. Established instruments were scored according to standard protocols, and exploratory descriptive statistics were calculated. Exploratory analysis was performed with paired *t*‐tests performed for scores between each time points and the *p* Value were determined accordingly. A *p* Value less than 0.05 was considered as statistically significant.

Outcomes were calculated for the percentage of patients who were able to complete (1) the MDASI, (2) the EQ‐5D‐5L (3) PROMIS4 after discharge. The percentage of patients who wore the pedometer was also assessed at each time point.

## 3 RESULTS

### 3.1 Sociodemographic and surgical characteristics

A total of 21 patients participated in the study; their sociodemographic characteristics are presented in Table 1. The median age was 58, 64% were male, 55% were of white race, 73% were married and 96% lived with a spouse/partner/friends, 36% lived with children and 27% were retired. The majority (76.2%) lived >15 miles away from the medical center.

**Table 1:** Sociodemographic characteristics

Variable	Value (*N* = 21 participants)
Age, median(range), year	58 (32−82)
Sex, *N* (%)	
Female	7 (32)
Male	14 (64)
Race, *N* (%)	
White	12 (55)
Black or African American	1 (5)
American Indian or Alaska Native	1 (5)
Asian	2 (9)
Other	4 (18)
Ethnicity, *N* (%)	
Hispanic or Latino	6 (27)
Non‐Hispanic	14 (64)
Marital status, *N* (%)	
Married	16 (73)
Domestic partnership	4 (18)
Separated	1 (5)
Living Situation, *N* (%)	
Spouse	18 (82)
Children	8 (36)
Friends/significant other	3 (14)
Education, *N* (%)	
Did not complete high school	2 (9)
Vocational school	1 (5)
Some college	6 (27)
Graduated college	7 (32)
Completed graduate school	5 (23)
Employment status, *N* (%)	
Full‐time	9 (41)
Retired	6 (27)
Unemployed	2 (9)
Other	4 (18)
Annual income, *N* (%)	
Less than $15 000	1 (5)
$15 001−30 000	2 (9)
$30 001−50 000	0
$50 001−75 0000	4 (18)
$75 001−100 00	5 (23)
Greater than 100 000	10 (45)
Insurance, *N* (%)	
Medicare	9 (41)
Private	11 (50)
Distance from medical center	
<5 miles	0
5−10 miles	2 (9.5)
11−15 miles	3 (14.3)
>15 miles	16 (76.2)

For surgical characteristics (see Table 2), the patients were relatively high risk, with 17 patients (77%) with either an ASA III or IV classification. Patients with ASA III are characterized as having systemic disease that is not incapacitating and ASA IV are patients with incapacitating systemic disease that is a constant threat to life. The majority of patients (68%) had a minimally invasive surgery (either robotic or laparoscopic). Nine patients (43%) had combined surgeries that involved multiple organs (colorectal + genitourinary or colorectal + liver). The median length of hospital stay was 7 days (range 0−36). The 30‐day readmission rate was 33%, with 7 patients returning for hospitalization.

**Table 2:** Surgical characteristics

Variable	Value (*N* = 21 participants)
ASA, *N* (%)	
II	1 (5)
III	9 (41)
IV	8 (36)
Comorbidities, *N* (%)	
Hypertension	9 (41)
Coronary artery disease (CAD)	2 (9)
Surgical Technique, *N* (%)	
Open	6 (27)
Laparoscopic or robotic assisted	15 (68)
Preoperative chemotherapy	7 (32)
Preoperative radiation therapy	2 (9)
Length of hospital stay, median (range)	7 (0−36)
Comprehensive complication index, median	9.5 (0–37.1)
Complications	11 (52.4)
Grade I	5 (23.8)
Grade II	1 (4.8)
Grade IIIa	5 (23.8)
Readmission within 30 day, *N* (%)	7 (33.3)
Resection site, *N* (%)	
Colorectal	3 (14.3)
Pancreas	2 (9.5)
Liver	1 (4.8)
Genitourinary (bladder and kidney)	4 (19)
Colorectal + genitourinary	7 (33.3)
Colorectal + liver	2 (9.5)

### 3.2 Feasibility

Feasibility was assessed by accrual, attrition and the ability of patients to use the equipment and the staff ability to act on alerts to threshold health care parameters. A total of 28 patients were identified and invited to participate in the study over a 4‐month period (7 patients per month). The accrual rate was 78% with 22 patients that agreed to participate and provided written informed consent. The most common reasons patients gave for declining participation were not being tech savvy, being overwhelmed/stressed, and already having pre‐arranged home services. One patient consented but subsequently withdrew due to reports of feeling overwhelmed, yielding a final sample of 21 patients with evaluable data and yielding an attrition rate of 4.5% (1/22).

Following informed consent, 20 out of the 21 patients (95%) completed the preoperative baseline PGHD assessment (vital signs and electronic surveys). At discharge, 91% (19/21) completed the assessments. At post‐discharge Days 2, 7, 14, and 30, the adherence rate for remote assessment completion was 82%, 68%, 64%, and 64%, respectively. The patients ability to complete the entirety of the study was 64% speaking to feasibility at 30 days postdischarge.

Overall, we observed high to acceptable levels of adherence with wearing the Vivofit pedometer. Before surgery, 18 out of 21 patients (85.7%) wore the Vivofit pedometer; the median of wear days was 6 (range 1−29 days). During postop hospitalization, 18 out of 21 (85.7%) wore the pedometer; the median number of wear days while hospitalized was 7 (range 2−36 days). At Days 2, 7, 14, and 30 postdischarge, the percentage of patients that wore the pedometer was 85.7%, 85.7%, 85.7%, and 61.9%. The median number of wear days after discharge was 35 (range 3−51 days). Thus feasibility as measured by patients ability to use the Vivofit varied with days from discharge as noted above indicating 85.7% up to 2 weeks postdischarge.

**Figure 1:**
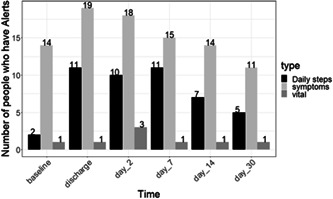
Vital sign and symptom alerts. The total number of patients with vital sign, step or ePROs alerts (Y axis on the left) and the study day (X axis). ePROs, electronic patient‐reported outcomes.

Findings on patients with triggered alerts types over time are depicted in Figure 1. The greatest number of vital sign alerts occurred on Day 2 postdischarge with three patients having pulse oximetry triggered alerts. For ePROs (symptom alerts), the greatest number of patients with alerts also occurred at Day 2 postdischarge with 18/21 (85.7%) patients generating some form of alert. A total of 200 ePRO alerts were triggered at that postdischarge Day 2 assessment time point. The most common and significant complaints related to ePRO alerts on Day 2 after discharge were secondary to mobility, self‐care, participation in usual activities, and loss of appetite (*p* < 0.05). It should be noted that over time the number of alerts generated for steps and symptoms trended down by Day 30.

### 3.3 Acceptability

At the end of the pilot, 72% (15/21) patients completed the satisfaction survey. Of those completing the survey, 80% felt the Vivofit watch was easy/extremely easy to use and 67% felt it was helpful to monitor daily activities. Overall 93% felt it was easy/extremely easy to use the devices to monitor blood pressure, weight, heart rate, temperature and oxygen levels and to complete the online surveys. The majority (73%) felty the length of the surveys was just right. Patients were also queried via open ended exit interviews on their subjective assessment of the perioperative telemonitoring program. The team was able to conduct exit interviews in 15 patients to obtain feedback on the perioperative monitoring. Of those interviewed, 87% (13/15) felt that the vital sign devices were helpful in monitoring how they were recovering physically. When asked if the devices helped them communicate their physical needs after surgery, 73% (11/15) said yes. Patient also felt that the pedometer was helpful in monitoring how they were recovering functionally (67%) and the vast majority felt that the online surveys were helpful in monitoring their symptoms and quality of life before and after surgery.

They were generally satisfied with the ability to monitor their vital signs and thus “eliminate a nurse coming” amidst the COVID‐19 pandemic. There were comments pertaining to the ease of use after overcoming the lack of familiarity with technology. Additionally, the most frequent comments were centered on the patient's ability to independently track their progress as it pertains to mobility, well‐being and pain assessment.

### 3.4 Trajectory of vital signs and daily steps overtime

Patient Generated Health data in the form of vital signs does not comprehensively reflect the patients perioperative experience or recovery. In this pilot study of 21 patients we did not see a trajectory of vital signs that changed over the postdischarge period and thus no definitive conclusions can be made from this aspect of the data.

**Figure 2:**
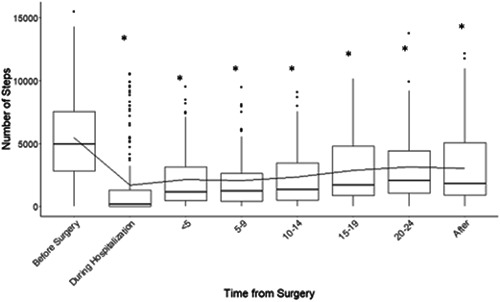
Steps (y axis) in the perioperative period. Data shown in intervals of time before surgery, during hospitalization and at 5 day intervals from discharge. Median number of steps before surgery (4957), during hospitalization (178), postdischarge days <5 (1142), 5−9 (1216), 10−14 (1345), 15−19 (1710), 20−24 (2074), 25−30 (after) (1802).

Daily steps trajectory over time is depicted in Figure 2. The median daily step count before surgery was 4957 (range 0−15 484); this number dropped to 178 (range 0−10 577) during hospitalization. A gradual increase in the number of daily steps were observed after Day 14 postdischarge. Overall, the number of daily steps did not reach baseline levels by Day 30 postdischarge.

Exploratory analysis on potential trends in functional declines and recovery found that patients took significantly fewer steps during hospitalization than baseline (before surgery); this trend persisted up to Day 14 postdischarge. There were no significant differences between the number of daily steps at baseline and those after Day 14 postdischarge.

### 3.5 ePRO trajectories overtime

Symptoms, QOL, and physical/mental health status score trajectories are presented in Table 3. In the Quality of Life Health Dimensions on a Scale of 0−5 (higher scores indicating worse problems), there was significant worsening of mobility, self‐care and usual daily activity at Day 2 postsurgery compared to baseline before surgery (*p* < 0.05). The challenges in usual activities persisted thought Day 14. As it pertains to the overall symptom scores on a Scale of 0−10 with higher scores indicating worse symptoms, Day 2 was significantly worse than before surgery (*p* < 0.05). As it pertains to individual symptom scores, the only value that was significant at discharge and at Day 2 postdischarge was appetite loss (*p* < 0.05). These results indicate that patients are most vulnerable and symptomatic at Day 2 postdischarge. This implies that Day 2 postdischarge is an opportunity to reach out to patients to intervene and address any symptoms, concerns or questions. These improved from postdischarge Day 7 to 30 and in many circumstances, the symptoms at Day 30 after discharge, were less than the before surgery time point. Overall, the symptoms that most often triggered an alert included pain, fatigue, sleep disturbance, appetite loss and distress.

**Table 3:** Symptoms and quality of life score

Variable	Mean (SD)					
	Before surgery	At discharge	Postdischarge			
			Day 2	Day 7	Day 14	Day 30
Quality‐of‐life health dimensions on a scale of 0−5, higher scores indicate more problems						
Mobility	1.8 (1.1)	2.3 (1)	**2.5** * (**0.8)**	2.1 (1)	2.3 (0.8)	1.7 (0.7)
Self‐care	1.4 (0.9)	2.0 (0.9)	**2.4** ** (**1.1)**	1.9 (0.9)	1.9 (1)	1.5 (0.7)
Usual activities	1.8 (1.8)	2.4 (1.2)	**3.1** *** (**0.9)**	**2.6** * (**1.1)**	**2.9** ** (**0.9)**	2.1 (0.7)
Pain or discomfort	2.0 (1.3)	2.6 (0.9)	3.0 (0.8)	2.7 (0.9)	2.6 (0.7)	2.2 (0.6)
Anxiety or depression	2.1 (1)	2.1 (1.1)	2.1 (1.1)	1.9 (0.9)	2.1 (0.9)	2.0 (0.8)
Overall symptom scores on a scale of 0−10, with higher scores indicating worse symptoms						
Symptom interference with activities	3.3 (3.3)	4.2 (3.5)	**5.1** * (**3.3**)	4 (2.8)	4.2 (2.9)	2.5 (2.3)
Symptom severity	2.3 (2.8)	2.8 (2.5)	2.7 (2.6)	2.8 (2.7)	2.5 (2.4)	1.6 (0.1)
Individual symptom scores on a scale of 0−10, with higher scores indicating worse symptoms						
Pain	3 (4.1)	4.2 (2.7)	4.2 (3.1)	4.5 (2.9)	4.2 (3)	2.4 (1.6)
Fatigue	3.5 (3.2)	5 (3)	4.5 (3)	4.8 (2.9)	4.2 (2.8)	3.4 (2.5)
Nausea	1.5 (2.5)	0.6 (1.6)	1 (2.1)	1.1 (2.4)	0.8 (1.7)	0.4 (0.9)
Sleep disturbance	3 (3.2)	5.8** (3)	4.3 (3.4)	4.5 (3.3)	3.9 (3.1)	2.9 (2.6)
Distress	3 (3)	2.8 (2.7)	3.6 (3.6)	3.5 (3)	3.2 (3)	2.1 (2)
Dyspnea	1.1 (2.3)	1.3 (2.5)	1.8 (2)	1.6 (2)	1.4 (1.7)	0.7 (1.1)
Memory	2.3 (2.7)	1.7 (2.3)	1.5 (1.6)	1.7 (2.0)	1.7 (1.7)	0.9 (1.3)
Appetite Loss	1.7 (2.6)	**3.7** * (**3.0)**	**3.6** * (**2.7)**	3.2 (2.9)	2.9 (3.2)	1.6 (1.4)
Drowsiness	2.8 (3.2)	3.5 (2.7)	2.7 (2.5)	3.6 (3.3)	2.7 (2.5)	2.1 (1.7)
Sadness	2.8 (3)	2.3 (3)	3 (3.5)	2.3 (2.4)	2.9 (3)	2 (1.9)
General health status on a scale of 0−100, with higher scores indicating better health						
Today's health score	57.1 (27.8)	63.5 (19.8)	49 (18.9)	61.1 (21.1)	60 (22.2)	62.9 (24.6)

Abbreviation: SD, standard deviation.

*p < 0.05, **p < 0.01, ***p < 0.001.

### 3.6 PGHDs and postoperative complications

**Figure 3:**
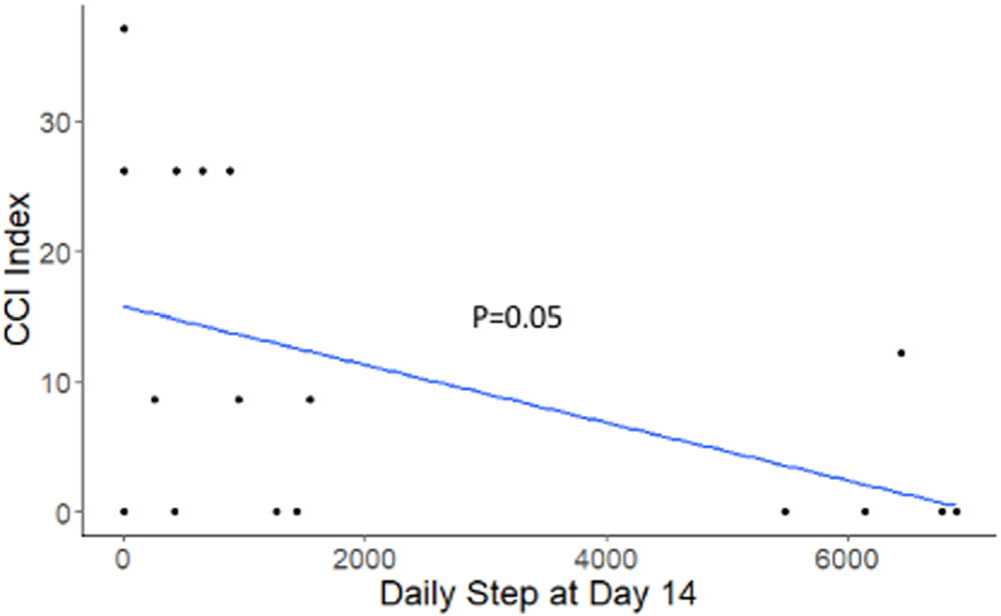
Association between comprehensive complication index and the number of daily steps at postoperative Day 14. Postoperative Day 14 daily step count best correlated with the CCI in this group of 20 patients with evaluable data. *p* = 0.05 and median overall CCI was 9.5 (range 0−37.1). CCI, comprehensive complication index.

We conducted exploratory analysis to understand the potential relationships between daily steps/functional recovery and postoperative complications. In Figure 3, we do not see a significant relationship between the number of daily steps at Day 14 postdischarge and postoperative complications (as measured by the Comprehensive Complication Index—CCI). These findings were the same for all of the postdischarge time points.

**Figure 4:**
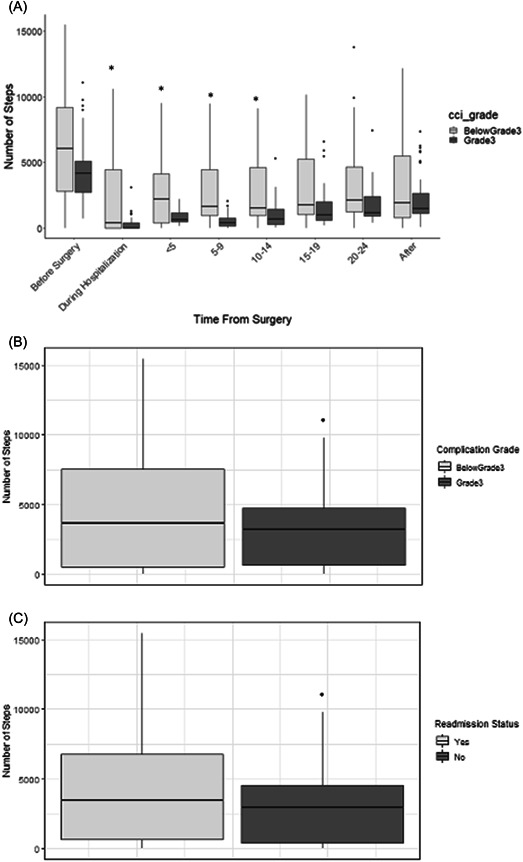
Steps (y axis) in the perioperative period and the presence or absence of ≥Grade 3 complications. There was a significant association between number of steps and the presence of a Grade 3 or higher complication at the following time intervals: before surgery, during hospitalization, less than 5 days postdischarge, between Days 5–0 postdischarge and at Days 10–14 postdischarge (*p < 0.05).

To assess if there was a difference in mobility based on the presence or absence of significant complications, we looked at patients step counts dichotomized by the ultimate development of a Grade 3a or higher complication at time points before surgery, during hospitalization, and at 5 day intervals after discharge. We found significant differences between the two groups before surgery, during hospitalization, up to Day 5 after discharge, Days 5−9, and Days 10−14 (Figure 4A). The median number of daily steps before surgery in the patients that had no complications or less than grade 3 was 6062 versus 4166 in patients that developed a complication that was grade 3 or higher (*p* < 0.05). These results imply that the mobility of the patient preoperatively may be associated with a higher grade of postoperative complications. It should also be noted that on the day before discharge there was a significant number of fewer steps taken by patients that developed ≥Grade 3 complications compared to those that had no complications or <Grade 3 morbidity (3186 [0−11066] vs. 3655 [0−15484], *p* < 0.05; Figure 4B). Similarly, on the day before discharge, patients that were ultimately readmitted took significantly fewer steps than patients who were not readmitted (2969 [0−15484] vs. 3469 [0−11066], *p* < 0.05; Figure 4C). These results indicate that degree of mobility on the day before discharge is associated with both more severe complications and readmissions and these patients may need consideration for more careful postdischarge monitoring.

## 4 DISCUSSION

In this study we demonstrate that perioperative telemonitoring in complex surgical oncology patients is feasible with adherence rates up to 64% at 30 days after discharge. We also demonstrate that the greatest alerts generated by patients are on postdischarge Day 2; highlighting this as a critical time point to monitor and intervene in the perioperative setting. Patients may be counseled that Day 2 postdischarge is most significant in terms of symptoms. By Day 30 postdischarge, patients should be back to baseline as it pertains to symptoms and in some cases improved from baseline.

Interestingly, we find that patient mobility the day before discharge correlates with ≥Grade 3a complications and readmissions. This identifies a patient population that needs more careful postdischarge perioperative monitoring to mitigate readmissions and severity of complications. As this was not a causality study, we are not able to discern whether the complication led to the lack of mobility. Additionally, mobility postdischarge is most impaired up to 2 weeks. After Day 14 postdischarge, patients steadily improve their activity and this time frame can be used to counsel and educate patients for postdischarge expectations in mobility improvements.

More than 45 million Americans undergo surgery each year, with expenditures exceeding $500 billion (40% of national health care spending).^17^ Expenditures for cancer care, including surgery, were $127 billion in 2013; this cost is projected to increase to $158 billion in 2020.^18^ More than 60% of cancer patients undergo surgical interventions,^19^ and surgery is often used as either the sole treatment modality or in combination with radiation and/or chemotherapy. Surgical interventions account for the most cures after a cancer diagnosis.^20^ To mitigate these costs, various approaches have been utilized to address these challenges. This study did not address potential cost savings however, this will be a critical metric in future work to assess the role of telemonitoring in this perioperative setting.

In recent years, surgical care is increasingly focused on using enhanced recovery after surgery (ERAS) pathways to improve surgical outcomes.^21^ ERAS pathways are created to include standard, prescribed tasks in the perioperative care setting to shorten length of hospital stay and contain cost.^22^ Perioperative care provided through ERAS pathways should also include remote monitoring and real‐time interventions after hospital discharge and until full postsurgical recovery. The concept is to identify postdischarge impending complications before them escalating and thus mitigate them and decrease readmissions. In this study we found that patients had the greatest aberrations in their vital signs, specifically their oxygenation at 2 days after discharge. Additionally, we found that the greatest concerns as they pertain to symptomatology and quality of life were at Day 2 postdischarge. By 30 days, these had all returned to baseline ePROs pertaining to both symptoms and quality of life and in many categories, these were actually better than baseline. These perhaps are a reflection of symptoms alleviated by the surgery itself or by the anxiety and concern of the uncertainty that may be associated with an upcoming surgery or recovery.

The past 50 years have seen an explosion in biomedical knowledge, dramatic innovations in surgical procedures, and management of complex medical conditions, with ever more exciting clinical capabilities on the horizon. Yet, the American health care system is falling short on key components of quality, outcomes, and cost.^23^ The overarching imperatives for health care include the need to develop ways to manage its ever‐increasing complexity, and curb ever‐escalating costs. Opportunities now exist to address these problems; these include (1) computational power that is affordable and widely available; (2) connectivity that allows information to be accessed in real‐time virtually anywhere; (3) human and organizational capabilities that improve the reliability and efficiency of care processes; (4) the recognition that effective care must be delivered in a patient‐centric fashion; and (5) the recognition that, regardless of incentive structures, penalties, and payment reforms, nothing about the experiences and outcomes/value of care will improve until progress is made to revolutionize the care delivery system.^18^, ^23^, ^24^ Telemonitoring in the perioperative period allows for this. In particular we were able to demonstrate that this method of interacting with the patients is acceptable and helpful in their subjective assessment of aiding in functional, symptom focus and quality of life arenas.

It should be noted that as the primary outcome measure in this study was feasibility, there was a clear decrease of adherence as the study progressed to postdischarge data. Several factors may contribute to this. In qualitative interviews of the participants and staff, these included challenges with the technology, recovery from complications and decreased motivation as recovery progressed. In future work it will be important to address these challenges with additional patient support in this perioperative window. Our findings of 30‐day adherence in the 64% range have been seen in other studies including the recently published Post‐discharge after surgery Virtual Care with Remote Automated Monitoring‐1 (PVC‐RAM‐1) trial.^25^

Our partnership with an existing home health monitoring and digital patient engagement infrastructure (mTelehealth™) leveraged a “real world” platform, which enhanced the successful implementation of this study and the future adoption into clinical surgical oncology practice. It should be noted that there are a multitude of digital patient engagement technologies. The agreement with mTelehealth was purely in the context of answering a research question with no financial relationship by any of the authors. Our study design moved away from traditional clinic‐based care paradigms to telehealth patient engagement. Most interestingly, we were able to show in this small pilot that patients who ultimately developed complications in the postoperative setting demonstrated significantly less mobility as measured by daily steps in the day before discharge. This implies that preoperative mobility is a potential predictor of outcomes and should be factored into the counseling and patient selection of patients planned for these complex oncologic procedures. The challenges of the United States health care system demand an innovative, transformative approach to perioperative and post‐discharge care. A great deal of further work is needed to optimize the modality, frequency, and mechanisms of comprehensive patient‐centric telemonitoring as an adjunct to traditional health care delivery.^23^

### 4.1 Limitations

There were several limitations of this study. First, there were only 21 analyzable patients in this pilot. The knowledge gained with this study will inform future prospective trials. Second, was the potential for over‐testing given over 50 variables and over 100 tests for 21 patients. In designing this pilot, patient burden is a critical consideration in ePROs and PGHD. However, in this study the tools utilized have been validated and at the set intervals are of reasonable burden spanning up to 7−10 min. The addition of nursing for technical support was there to mitigate some of this burden. This compilation of vital signs, ePROs and mobility data is critical to be done simultaneously to inform what aspects of telemonitoring is the most valuable. This data can then be utilized in larger multicenter trials. The recently published PVC‐RAM‐1 trial failed to find a difference in survival in this context and that postoperative telemonitoring can be effectively carried in about 2/3 of complex surgical oncology patients.^25^ Our findings were similar in this pilot as it pertains to adherence for the duration of the 30 days postdischarge.

Third, in evaluating the presence of Grade 3A or higher complications, we are not able to retrospectively assess risk factors in a multivariable analysis in this 21 patient pilot. Additionally, we were not able to monitor patients 24 h a day, 7 days a week in a continuous fashion thus potentially missing changes in vital signs that may have correlated with complications or readmissions. We were not able to complete exit interviews in all 21 patients in the study and thus perhaps are missing important insight from the 6 remaining participants. Last, it should be acknowledged that all analyses in this pilot are exploratory and hypothesis generating.

## 5 CONCLUSIONS

Telemonitoring and telehealth are a perioperative modality of care that will surely be incorporated as we improve our digital interface with patients once they leave the hospital. In this study we demonstrate that this is a feasible and acceptable approach. Future work is needed to assess which aspects and of what duration does telemonitoring render the most value to patients and health systems to optimize outcomes and minimize resource utilization.

## CONFLICT OF INTEREST

The authors declare no conflicts of interest.

## SYNOPSIS

The feasibility of remote perioperative telemonitoring of patient‐generated physiologic health data and patient‐reported outcomes in a high risk complex general and urologic oncology surgery population is evaluated. Future studies will ascertain optimal patient selection, duration, and extent of perioperative monitoring.

## Data Availability

The data that support the findings of this study are available on request from the corresponding author. The data are not publicly available due to privacy or ethical restrictions.
